# Fauna Europaea: Coleoptera 2 (excl. series Elateriformia, Scarabaeiformia, Staphyliniformia and superfamily Curculionoidea)

**DOI:** 10.3897/BDJ.3.e4750

**Published:** 2015-04-09

**Authors:** Paolo Audisio, Miguel-Angel Alonso Zarazaga, Adam Slipinski, Anders Nilsson, Josef Jelínek, Augusto Vigna Taglianti, Federica Turco, Carlos Otero, Claudio Canepari, David Kral, Gianfranco Liberti, Gianfranco Sama, Gianluca Nardi, Ivan Löbl, Jan Horak, Jiri Kolibac, Jirí Háva, Maciej Sapiejewski, Manfred Jäch, Marco Alberto Bologna, Maurizio Biondi, Nikolai B. Nikitsky, Paolo Mazzoldi, Petr Zahradnik, Piotr Wegrzynowicz, Robert Constantin, Roland Gerstmeier, Rustem Zhantiev, Simone Fattorini, Wioletta Tomaszewska, Wolfgang H. Rücker, Xavier Vazquez-Albalate, Fabio Cassola, Fernando Angelini, Colin Johnson, Wolfgang Schawaller, Renato Regalin, Cosimo Baviera, Saverio Rocchi, Fabio Cianferoni, Ron Beenen, Michael Schmitt, David Sassi, Horst Kippenberg, Marcello Franco Zampetti, Marco Trizzino, Stefano Chiari, Giuseppe Maria Carpaneto, Simone Sabatelli, Yde de Jong

**Affiliations:** ‡ Sapienza Rome University, Department of Biology and Biotechnologies 'C. Darwin', Rome, Italy; § Museo Nacional de Ciencias Naturales, Madrid, Spain; | CSIRO Entomology, Canberra, Australia; ¶ Umea University, Umea, Sweden; # National Museum Prague, Prague, Czech Republic; ¤ Queensland Museum, Brisbane, Australia; « Departamento de Biología Animal, Santiago de Compostela, Spain; » Unaffiliated, San Donato Milanese, Italy; ˄ Charles University, Prague, Czech Republic; ˅ Via Cascina Girola, Uboldo, Italy; ¦ Via Raffaello Sanzio 84, Cesena, Italy; ˀ MiPAAF, Corpo Forestale dello Stato, Centro Nazionale per lo Studio e la Conservazione della Biodiversità Forestale “Bosco Fontana” di Verona, Sede di Bosco Fontana, Strada Mantova 29, I-46045, Marmirolo (MN), Italy; ˁ Museum d'Histoire naturelle Geneve, Geneve, Switzerland; ₵ K Hádku 1567, Dubeček, CZ-107 00 Praha 10, Prague, Czech Republic; ℓ Moravian Museum, Brno, Czech Republic; ₰ Dermestidae World, Prague, Czech Republic; ₱ Museum and Institute of Zoology, Warsaw, Poland; ₳ Naturhistorisches Museum Wien, Wien, Austria; ₴ Department of Sciences, University Roma Tre, Roma, Italy; ₣ University of L`Aquila, Department of Health, Life and Environmental Sciences, L`Aquila - Coppito, Italy; ₮ Moscow State University, Moscow, Russia; ₦ private, Via G. Galileo 87, Brescia, Italy; ₭ Forestry and Game Management Research Institute, Praha, Czech Republic; ₲ Unaffiliated, Saint-Lo, France; ‽ Technische Universitaet Muenchen, Freising, Germany; ₩ C/O Zoological Museum, Sapienza Rome University, Rome, Italy; ₸ Von-Ebner-Eschenbach-Straße 12, Neuwied, Germany; ‡‡ University of Barcelona, Barcelona, Spain; §§ Via Fulvio Tomassucci 12/20, I-00144, Rome, Italy; || private, Francavilla Fontana (BR), Italy; ¶¶ The Manchester Museum, Manchester, United Kingdom; ## Staatliches Museum fuer Naturkunde, Stuttgart, Germany; ¤¤ Università degli Studi di Milano, Milano, Italy; «« University of Messina, Department of Biological and Environmental Sciences, Messina, Italy; »» University of Florence, Natural History Museum, Zoological Section 'La Specola', Florence, Italy; ˄˄ Institute of Agroenvironmental and Forest Biology, CNR - National Research Council of Italy, Monterotondo Scalo (Rome), Italy; ˅˅ Naturalis Biodiversity Center, Leiden, Netherlands; ¦¦ Ernst Moritz Arndt Universitaet, Greifswald, Germany; ˀˀ c/o Museo Civico di Storia Naturale, Milano, Italy; ˁˁ private, Langer Platz 21, D – 91074 Herzogenaurach, Germany; ₵₵ Department of Genetics, University of Pennsylvania, Philadelphia, United States of America; ℓℓ Department of Sciences, University Roma Tre, Rome, Italy; ₰₰ University of Eastern Finland, Joensuu, Finland; ₱₱ University of Amsterdam - Faculty of Science, Amsterdam, Netherlands

**Keywords:** Biodiversity Informatics, Coleoptera, Fauna Europaea, Taxonomic indexing.

## Abstract

Fauna Europaea provides a public web-service with an index of scientific names (including synonyms) of all living European land and freshwater animals, their geographical distribution at country level (up to the Urals, excluding the Caucasus region), and some additional information. The Fauna Europaea project covers about 230,000 taxonomic names, including 130,000 accepted species and 14,000 accepted subspecies, which is much more than the originally projected number of 100,000 species. This represents a huge effort by more than 400 contributing specialists throughout Europe and is a unique (standard) reference suitable for many users in science, government, industry, nature conservation and education.

Coleoptera represent a huge assemblage of holometabolous insects, including as a whole more than 200 recognized families and some 400,000 described species worldwide. Basic information is summarized on their biology, ecology, economic relevance, and estimated number of undescribed species worldwide. Little less than 30,000 species are listed from Europe. The Coleoptera 2 section of the Fauna Europaea database (Archostemata, Myxophaga, Adephaga and Polyphaga excl. the series Elateriformia, Scarabaeiformia, Staphyliniformia and the superfamily Curculionoidea) encompasses 80 families (according to the previously accepted family-level systematic framework) and approximately 13,000 species. Tabulations included a complete list of the families dealt with, the number of species in each, the names of all involved specialists, and, when possible, an estimate of the gaps in terms of total number of species at an European level. A list of some recent useful references is appended. Most families included in the Coleoptera 2 Section have been updated in the most recent release of the Fauna Europaea index, or are ready to be updated as soon as the FaEu data management environment completes its migration from Zoological Museum Amsterdam to Berlin Museum für Naturkunde.

## Introduction

In 1998 the European Commission published the European Community Biodiversity Strategy, providing a framework for the development of Community policies and instruments to comply with the Convention on Biological Diversity. The Strategy recognises the current incomplete state of knowledge at all levels concerning biodiversity, which is a constraint on the successful implementation of the Convention. *Fauna Europaea* contributes to this Strategy by supporting one of the main themes: to identify and catalogue the components of European biodiversity into a database to serve as a basic tool for science and conservation policies. In regard to biodiversity in Europe, science and policies depend on the knowledge of its components. Biodiversity assessments, monitoring changes, sustainable exploitation of biodiversity, and much legislative work depend upon a validated overview of taxonomic biodiversity, in which *Fauna Europaea* plays a major role, providing a web-based information infrastructure with an index of scientific names (including important synonyms) of all living European land and freshwater animals, their geographical distribution at country level and some additional optional information (like references and species annotations). Thus the Fauna Europaea database provides a unique reference for many user-groups such as scientists, governments, industries, conservation communities and educational programs.

Fauna Europaea (FaEu) began in 2000 as an EC-FP5 four year project, delivering its first release in 2004 (de Jong et al. 2014). After thirteen years of steady progress to efficiently disseminate Fauna Europaea results and to increase the acknowledgement of the Fauna Europaea contributors, novel e-Publishing tools have been applied to prepare data papers of all major taxonomic groups (see below).

Most families included in the Coleoptera 2 Section (ca. 13,000 species) have been updated in the most recent release of the Fauna Europaea index, or are ready to be updated as soon as the FaEu data management environment completes its migration from Zoological Museum, Amsterdam to Berlin Museum für Naturkunde. Adopted systematics follows that used in the first release of the database (2004). Recent changes in family-level systematics of beetles introduced by Bouchard et al. 2011 (although not all were accepted by specialists) are foreseen to be implemented as soon as the FaEu data management environment completes its migration. For example, in *Fauna Europaea* the classic treatment of Chrysomelidae Galerucinae and Alticinae as separate subfamilies was used instead of the current view of Alticini as a tribe in Galerucinae, and the current families Megalopodidae and Orsodacnidae are not used, the European species being listed in subfamilies Zeugophorinae and Orsodacninae. The same is true for some other families which changed for different reasons their present-day taxonomic rank (e.g., Anobiidae vs. Ptinidae, Carabidae Rhysodinae vs. Rhysodidae, etc.).

### Data-papers & gap-analysis

To improve the dissemination and citation of Fauna Europaea and to increase the acknowledgement of the Fauna Europaea contributors, a special Biodiversity Data Journal (BDJ) Series has been compiled, using novel e-Publishing tools, called Contributions on Fauna Europaea, preparing data-papers of all major Fauna Europaea taxonomic groups. This work was initiated during the ViBRANT project and is further supported by the recently started EU BON project. This paper represents the first publication of the Fauna Europaea Coleoptera (excl. Elateriformia, Scarabaeiformia, Staphyliniformia) data sector as a BDJ data paper.

Further steps will be made on implementing Fauna Europaea in the EU BON project as a basic tool and standard reference for biodiversity research in Europe, and to evaluate the status of European taxonomic expertise. The Fauna Europaea data-papers will contribute to a quality assessment on biodiversity data by providing estimates on gaps in taxonomic information and knowledge (see Table 1).

## General description

### Purpose

Fauna Europaea is a database of the scientific names and distribution of all living, currently known multicellular European land and fresh-water animal species assembled by a large network of experts. An extended description of the Fauna Europaea project can be found in de Jong et al. 2014. A summary is given in the sections below.

**Figure 1.**
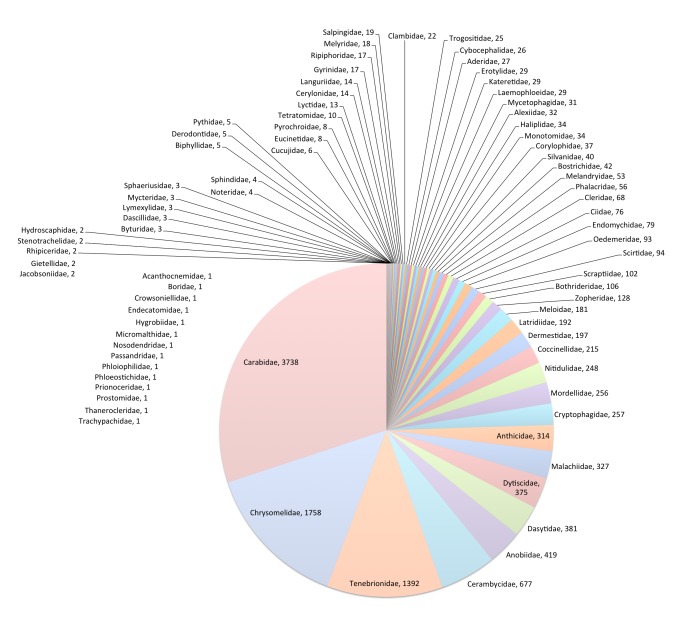
FaEu Coleoptera species per family. See Table 1 for family statistics. For full resolution see Suppl. material 1.

Coleoptera is the largest of the 58 *Fauna Europaea* major taxonomic groups, covering nearly 29,000 species in Europe [its Coleoptera 2 Section includes > 13,000 species (Fig. 1) and is represented by a network of more than 40 specialists (Table 1)].

### Additional information

**Coleoptera** [Group Coordinators: Paolo Audisio (Coleoptera 2), Miguel Angel Alonso-Zarazaga (Coleoptera 1)]

Coleoptera are the most diverse order of all living animals, and comprise between 360,000 and 400,000 named species worldwide (Chapman 2009; Slipinski et al. 2011; Zhang 2013; Audisio unpublished data), some 100,000 in the Palaearctic Region, and nearly 30,000 in European-Mediterranean areas. Beetles are the dominating insect group in all terrestrial environments, with the single exception of freshwater habitats, where Diptera are represented by a markedly larger number of species. Even using a conservative estimate, there are likely one to three million beetle species on the Earth. Coleoptera are ecologically diverse (Crowson 1981). Most members of the largest ‘basal’ suborder, Adephaga, are predatory in both the larval and imaginal stage, while most members of the huge suborder Polyphaga are phytosaprophagous, mycetophagous, predaceous, phytophagous, or xylophagous. The ‘basal’ suborder Archostemata is represented by a small number of families and species, mostly distributed in tropical areas, and usually associated with saproxylic habitats. The only known W Palaearctic autochtonous species, *Crowsoniella relicta* Pace from central Italy, exhibits an unknown biology, but it was collected, only once, in hypogeous habitats among tree roots, in carbonatic soils (Ge et al. 2011). In the suborder Adephaga, the largest family is represented by Carabidae, almost all of them having a predaceous life style in terrestrial habitats (relatively few species are seed-eating or myrmecophilous), while other families (e.g., Dytiscidae) inhabit freshwater habitats, where they are mostly predators of other aquatic organisms, only the family Haliplidae includes phytophagous species. The problematic suborder Myxophaga, recently considered questionable from a phylogenetic point of view (Beutel and Haas 2000; Friedrich et al. 2009), is represented by relatively few species mostly associated with mud and wet habitats, chiefly in thermal localities. The huge suborder Polyphaga (including about 90% of Coleoptera worldwide) is a large assemblage of families where both adults and larvae exhibit very diverse life styles. Among members of the large ‘basal’ superfamily Staphylinoidea, there is a prevalence of predaceous beetles. About one-fifth of Staphylinidae however can be characterized as mycetophagous or saprophagous. A smaller part of them (about 10% of European species) may be characterized as phytophagous or myrmecophilous. Most Staphylinoidea are terrestrial, but in a few families (e.g., Hydraenidae) nearly all species are adapted to an aquatic or semi-aquatic life style, even in very peculiar habitats such as hyperhaline marine rock-pools (Antonini et al. 2010; Audisio et al. 2010; Sabatelli et al. 2013). Most Elateroidea are predators, xylosaprophagous, or xylophagous. Cucujoidea are a large and highly diverse superfamily including species which are mostly saprophagous, mycetophagous, predaceous, phytophagous, or xylophagous, with a few families (e.g., Meloidae, Ripiphoridae) known as specialized parasitoids of other insects (Bologna 1991; Bologna et al. 2008; Bologna et al. 2010; Bologna and Di Giulio 2011; Lawrence et al. 2010). Scarabaeoidea include thousands of species mostly associated with dung of vertebrates, or having rhyzophagous or xylosaprophagous larvae, whereas adults are mostly floricolous. Chrysomeloidea include thousands of species within the main families Chrysomelidae and Cerambycidae, mostly phyllophagous and/or floricolous, or with xylophagous larvae (Biondi et al. 2013;Bouchard et al. 2009). Finally, the most speciose superfamily Curculionoidea, feeding on various plant matter, includes many important pests of cultivated crops and forest habitats as well as some important biological control agents of invasive weeds too (e.g., Ceutorhynchini) (Alonso-Zarazaga and Lyal 1999; Oberprieler et al. 2007). About 29,000 species of Coleoptera are listed for Europe (including more than 4,000 Adephaga, and little less than 25,000 Polyphaga); the taxonomic composition of this fauna is far better known than that of any other major region. But the species numbers occurring in the Afrotropical, Indo-Malayan and Neotropical regions are markedly higher, each of them with an estimated number of 70-90,000 named species. Most families of Coleoptera (at least in the largest suborder Polyphaga) are, in fact, largely represented in tropical and subtropical countries. However, the number of species annually added to the European beetle fauna (including autochthonous species new to Science, or firstly discovered in Europe) is relatively constant over time, while the introduction of alien species is continuously increasing, chiefly among the guilds associated with fruit, timber, stored and cultivated products, and ornamental plants (DAISIE 2008). The species accumulation curve, as in other large groups of insects such as Diptera, shows no signs of levelling off (Fontaine et al. 2012; Audisio unpublished data). Among the Adephaga, the most species rich families in the European fauna are Carabidae and Dytiscidae, with nearly 3,800 and 400 species respectively. Among the Polyphaga, the most species rich families in the European fauna are Staphylinidae s.l. (ca. 6,000 species), Curculionidae (> 4,500 species), Chrysomelidae (ca. 1,700 species), Tenebrionidae (> 1,400 species), Leiodidae (ca. 1,200 species), Elateridae and Cerambycidae (ca. 700 species each), Cantharidae (> 500 species), Dytiscidae, Hydraenidae, and Buprestidae (> 400 species each). Much remains certainly to be discovered, because especially Curculionidae, Staphylinidae and some small groups (such as, e.g., Bothrideridae, Alexiidae) were poorly studied by modern taxonomists and are much more diverse than suggested by their current count. Coleoptera are among the most important agricultural pests, attacking all parts of living plants as well as stored products such as woody matter, processed fibers and grains (BUSS and Fasulo 2006). Some of them are among the most serious pests of of beehives (Marini et al. 2013), while other groups are active predators or parasitoids (e.g., Carabidae, Coccinellidae, Meloidae, Cleridae) and play a fundamental role in both natural and cultivated environments, as important biological controllers that regulate the number of aphids, scale insects, wood borer species and locusts. On the other hand, beetles are active decomposers and play a major role in recycling organic waste, chiefly vertebrate dung and carcasses, decaying fruit, fungi and dead wood in forest habitats. Many beetles are, in fact, saproxylic, and are considered excellent indicators of woodland quality (Speight 1989; Nieto and Alexander 2010), several being well-known indicators of old-growth forests. Some flagship- and/or umbrella-species of forest habitats are recognized among the large-sized Lucanidae, Cetoniidae, Cerambycidae, and Cucujidae, which also are target species for biodiversity conservation efforts, and priority species included in annexes II and IV of the EU Habitat Directive. Some of them, like the rare but popular *Osmoderma eremita*, drives most of the European and local policies on invertebrate conservation biology and forest management (Chiari et al. 2013, Chiari et al. 2014). Other beetles are excellent indicators of quality (Trizzino et al. 2013), and several studies have been aimed to the use of this group as a tool for river quality assessment, for the management of lotic ecosystems (Trizzino et al. 2015), and for the evaluation/prediction of Climate Change’s effects. Finally, the use of certain groups of terrestrial Coleoptera such as ground beetles (Carabidae) and darkling beetles (Tenebrionidae) in the evaluation of the biological quality of the soil is covered by a vast literature (Kotze et al. 2011).

Several species among those in Coleoptera 2 Section have been also included in European Red Lists, such as the recent (although markedly incomplete) IUCN Saproxylic Beetles Red List of Neto & Alexander (Nieto and Alexander 2010). A number of other national, local, and European red lists have been recently published or are in preparation, and the role of *Fauna Europaea* as a standard reference for all these initiatives is more and more evident. The same is true for a number of pest species, quarantine species, and alien species (chiefly in Nitidulidae, Chrysomelidae, Coccinellidae, Cryptophagidae, Cerambycidae, Curculionidae, and others), whose introduction into Europe, as discussed above, is continuously increasing (Buss and Fasulo 2006; DAISIE 2008; Baviera and Audisio 2014; Audisio et al. 2014).

As shown in Table 1, the taxonomic coverage of Coleoptera 2 Section of the FaEu database is generally good, with few remaining gaps (most of them should be filled in the next upcoming phase of data base updating, probably in Summer 2015). However, certain groups like Ciidae, Cybocephalidae, Cryptophagidae, Bothrideridae, Scraptiidae, and Mordellidae, need the activity of a larger number of specialists both in the field and in museum collections, in order to significantly improve our present-day knowledge in taxonomy and distribution, chiefly in the most potentially species-rich countries of southern Europe. Among the specialists' network, almost all explicitly or implicitly confirmed their participation to the project, although financial support to the project was interrupted some ten years ago. Only a couple of specialists resigned (e.g. in Hydroadephaga) and were replaced during the running activity of file updating, or have recently received the aid of "new" specialists and cooperators of the Group Coordinator PA. Generally speaking, the European network of specialists involved in the Coleoptera 2 Section of the *Fauna Europaea* Project seems to be relatively consolidated, and open to new (welcome) entries, although there is evidence that in most recent years the European beetle taxonomy community, chiefly at a professional level, has been going through a significant "crisis of vocations", only partially and insufficiently facilitated by the scientific support of a lot of (mostly not young) amateur entomologists (Fontaine et al. 2012). A more extensive and better addressed public financial support, at both European and local levels, should be foreseen in the next years, to prevent the risk of a future dramatic "taxonomic impediment" in the scientific management of European insect biodiversity.

## Project description

### Title

This BDJ data paper includes the taxonomic indexing efforts in Fauna Europaea on European Coleoptera covering the first two versions of Fauna Europaea worked on between 2000 and 2013 (up to version 2.6).

### Personnel

The taxonomic framework of Fauna Europaea includes partner institutes, providing taxonomic expertise and information, and expert networks maintaining data collation.

Every taxonomic group is covered by at least one Group Coordinator responsible for the supervision and integrated input of taxonomic and distributional data of a particular group. For Coleoptera 2 the responsible Group Coordinator is Paolo Audisio (versions 1 & 2).

The Fauna Europaea checklist would not have reached its current level of completion without the input from several groups of specialists. The formal responsibility of collating and delivering the data of relevant families has resided with the below appointed Taxonomic Specialists (see Table 1), while Associate Specialists deserve credit for their important contributions at various levels, including particular geographic regions or (across) taxonomic groups.

Data management tasks are taken care primarily by the Fauna Europaea project bureau. During the project phase (until 2004) a network of principal partners managed the diverse management tasks: Zoological Museum Amsterdam (general management & system development), Zoological Museum of Copenhagen (data collation), National Museum of Natural History in Paris (data validation) and Museum and Institute of Zoology in Warsaw (NAS extension). Since the formal project ending (2004-2013) all tasks have been taken over by the Zoological Museum Amsterdam.

### Study area description

**Figure 2.**
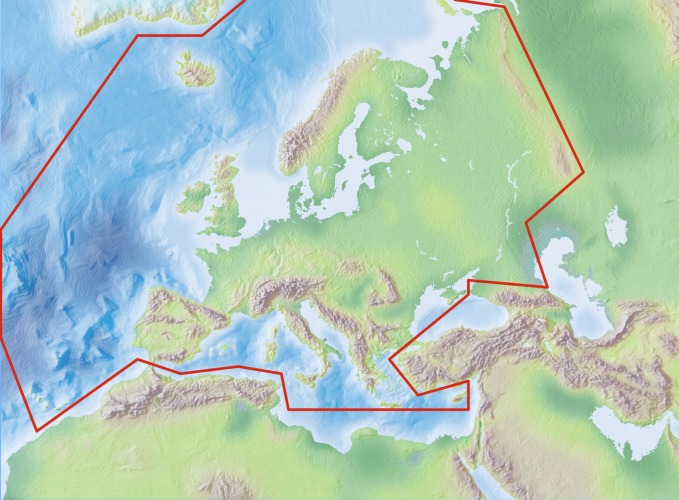
Fauna Europaea geographic coverage ('minimal Europe').

The area studied (Fig. 2) covers the European mainland (Western Palearctic), including the Macaronesian islands, excluding the Caucasus, Turkey, Arabian Peninsula and Northern Africa.

### Design description

Standards. Group coordinators and taxonomic specialists deliver the (sub)species names according to strict standards. The names provided by FaEu are scientific names. The taxonomic scope includes issues like, (1) the definition of criteria used to identify the accepted species-group taxa, (2) the hierarchy (classification scheme) for the accommodation of all accepted species and (3), relevant synonyms, and (4) the correct nomenclature. The Fauna Europaea 'Guidelines for Group Coordinators and Taxonomic Specialists', include the standards, protocols, scope, and limits that provide the instructions for all more then 400 specialists contributing to the project.

**Figure 3.**
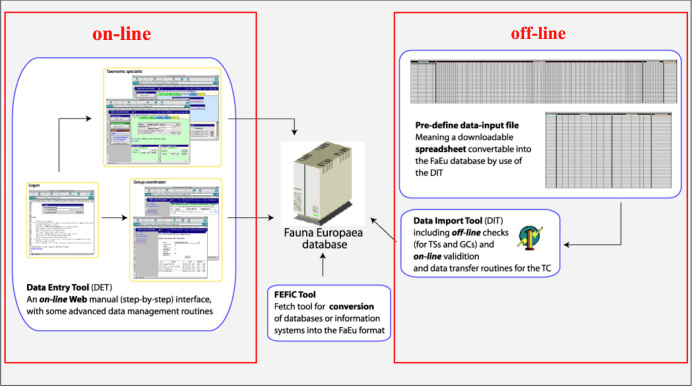
Fauna Europaea on-line (browser interfaces) and off-line (spreadsheets) data entry tools.

Data management. The data records could either be entered offline into a preformatted MS-Excel worksheet or directly into the Fauna Europaea transaction database using an online browser interface (see: Fig. 3). Since 2013, the data servers are hosted at the Museum für Naturkunde in Berlin.

Data set. The Fauna Europaea basic data set consists of: accepted (sub)species names (including authorship), synonyms (including authorship), taxonomic hierarchy / classification, misapplied names (including misspellings and alternative taxonomic views), homonym annotations, expert details, European distribution (at country level), Global distribution (only for European species), taxonomic reference (optional), and occurrence reference (optional).

### Funding

Fauna Europaea was funded by the European Commission under the Fifth Framework Programme and contributed to the Support for Research Infrastructures work programme with Thematic Priority Biodiversity (EVR1-1999-20001) for a period of four years (1 March 2000 - 1 March 2004), including a short 'NAS extension', allowing EU candidate accession countries to participate. Follow-up support was given by the EC-FP5 EuroCAT project (EVR1-CT-2002-20011), by the EC-FP6 ENBI project (EVK2-CT-2002-20020), by the EC-FP6 EDIT project (GCE 018340), by the EC-FP7 PESI project (RI-223806) and by the EC-FP7 ViBRANT project (RI-261532). Continuing management and hosting of the Fauna Europaea services was supported by the University of Amsterdam (Zoological Museum Amsterdam) and SARA/Vancis. Recently the hosting of Fauna Europaea was taken over by the Museum für Naturkunde in Berlin, supported by the EC-FP7 EU BON project (grant agreement №308454).

## Sampling methods

### Study extent

See spatial coverage and geographic coverage descriptions.

### Sampling description

Fauna Europaea data have been assembled by principal taxonomic experts, based on their individual expertise, including literature study, collection research, and field observations. No less than 476 experts contributed taxonomic and/or faunistic information for Fauna Europaea. The vast majority of the experts are from Europe (including EU non-member states). As a unique feature, Fauna Europaea funds were set aside for paying/compensating for the work of taxonomic specialists and group coordinators (around five Euro per species).

To facilitate data transfer and data import, sophisticated on-line (web interfaces) and off-line (spreadsheets) data-entry routines have been built, well integrated within an underlying central Fauna Europaea transaction database (see Fig. 3). This includes advanced batch data import routines and utilities to display and monitor the data processing within the system. In retrospect, it seems that the off-line submission of data was probably the best for bulk import during the project phase, while the on-line tool was preferred to enter modifications in later versions. This system works well until it supposed replacement in 2013.

A first release of the Fauna Europaea index via the web-portal has been presented at 27^th^ of September 2004. The most recent release (version 2.6.2) was launched at 29 August 2013. An overview of Fauna Europaea releases can be found here: http://www.faunaeur.org/about_fauna_versions.php.

### Quality control

Fauna Europaea data are unique in a sense that they are fully expert based. Selecting leading experts for all groups included a principal assurance of the systematic reliability and consistency of the Fauna Europaea data.

Further all Fauna Europaea data sets are intensively reviewed at regional and thematic validation meetings, at review sessions on taxonomic symposia (for some groups), by Fauna Europaea Focal Points (during the FaEu-NAS and PESI projects) and by various end-users sending annotations using the web form at the web-portal. Additional validation on gaps and correct spelling was effected at the validation office in Paris.

In conclusion, we expect to get taxonomic data for 99.3% of the known European fauna. The faunistic coverage is not quite as good, but is nevertheless 90-95% of the total fauna. Recognised gaps in Coleoptera includes some tribes of Staphylinidae, some minor tribes of Curculionidae, and a few minor families of Polyphaga, chiefly in SE Europe and in European Russia.

Checks on technical and logical correctness of the data have been implemented in the data entry tools, including around 50 "Taxonomic Integrity Rules". This validation tool proved to be of huge value for both the experts and project management, and significantly contribute(d) to preparation of a remarkably clean and consistent data set.

This thorough reviewing makes Fauna Europaea the most scrutinised data set in its domain.

### Step description

By evaluating team structure and life cycle procedures (data-entry, validation, updating, etc.), clear definitions of roles of users and user-groups, according to the taxonomic framework were established, including ownership and read and writes privileges, and their changes during the project life-cycle. In addition, guidelines on common data exchange formats and codes have been issued (see also the 'Guidelines for Experts' document).

## Geographic coverage

### Description

Species and subspecies distributions in Fauna Europaea are registered at least a country level, meaning political countries. For this purpose the FaEu geographical system basically follows the TDWG standards. The covered area includes the European mainland (Western Palearctic), plus the Macaronesian islands (excl. Cape Verde Islands), Cyprus, Franz Josef Land and Novaya Zemlya. Western Kazakhstan and the Caucasus are excluded (see Fig. 2).

The focus is on species (or subspecies) of European multicellular animals of terrestrial and freshwater environments. Species in brackish waters, occupying the marine/freshwater or marine/terrestrial transition zones, are generally excluded.

### Coordinates

Mediterranean (N 35°) and Arctic Islands (N 82°) Latitude; Atlantic Ocean (Mid-Atlantic Ridge) (W 30°) and Ural (E 60°) Longitude.

## Taxonomic coverage

### Description

**Figure 4.**
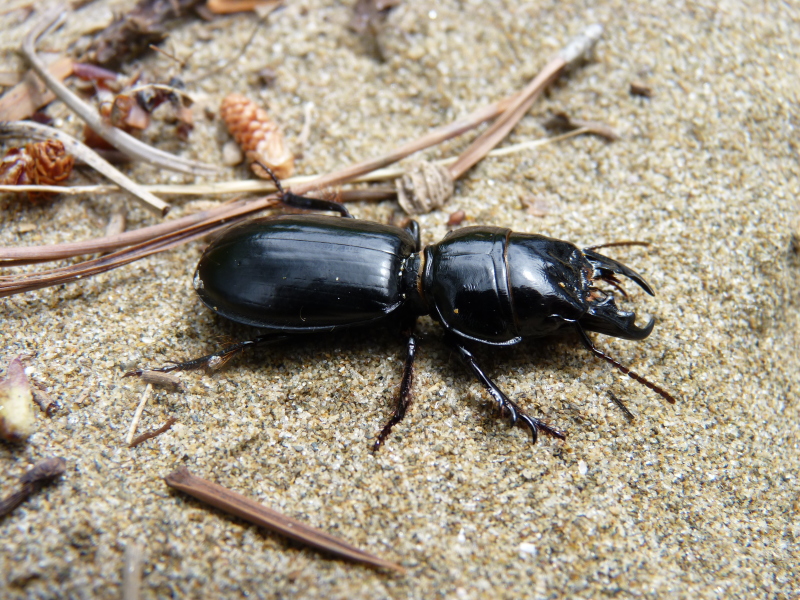
*Scarites buparius* (Forster, 1771) – Carabidae – photo by Paolo Audisio

**Figure 5.**
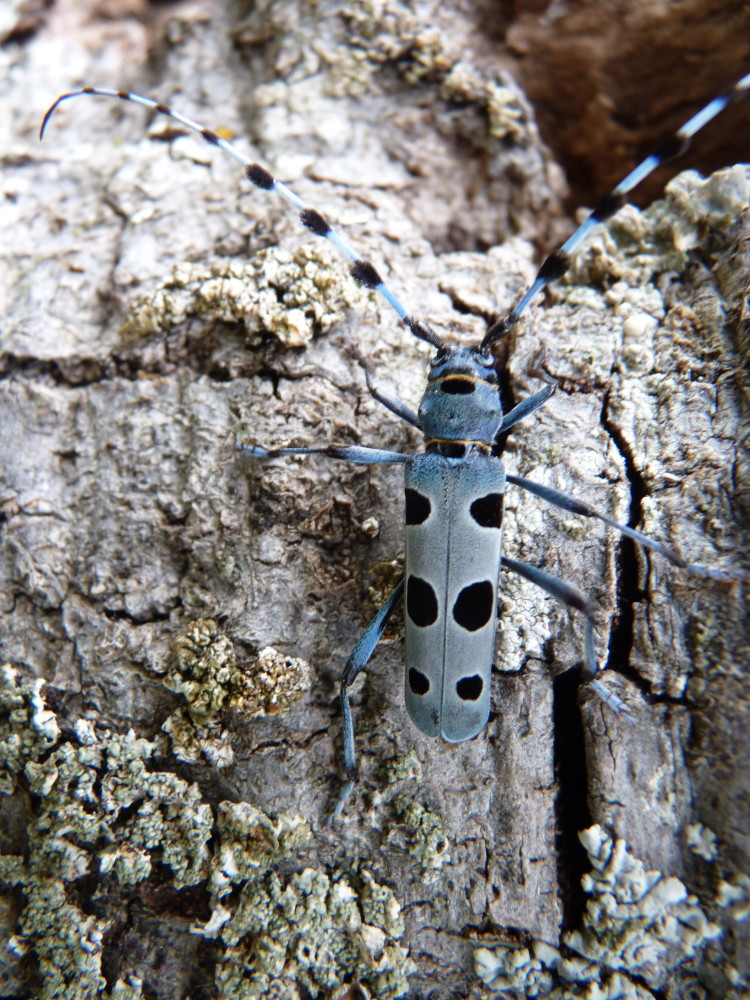
*Rosalia alpina* (Linnaeus, 1758) – Cerambycidae – photo by Paolo Audisio

**Figure 6.**
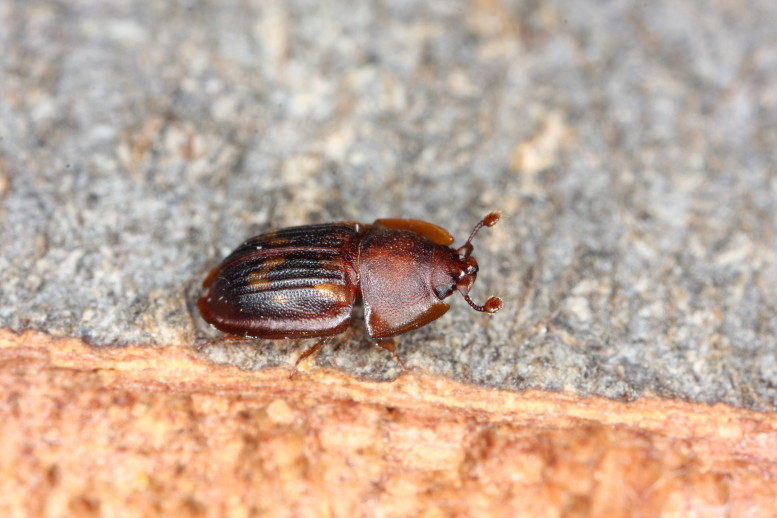
*Amphotis marginata* (Fabricius, 1781) – Nitidulidae – photo by Christoph Benisch – www.kerbtier.de

**Figure 7.**
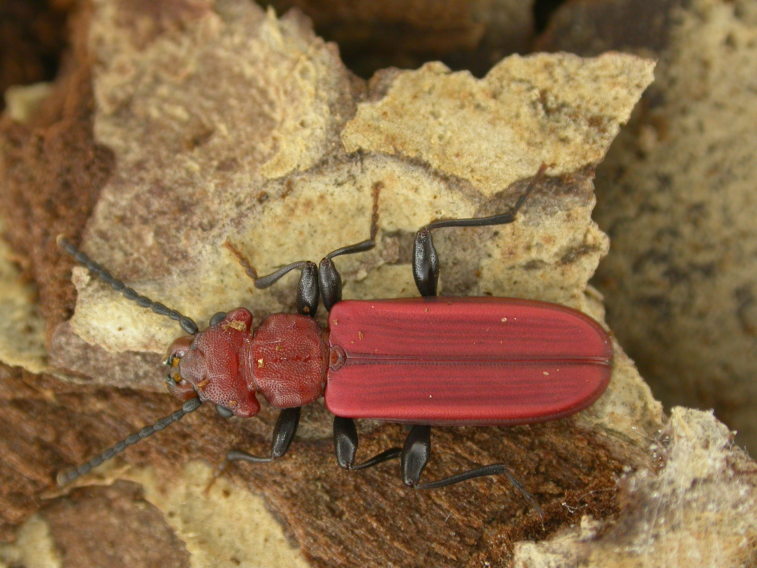
*Cucujus haematodes* Erichson, 1845 – Cucujidae – photo by Antonio Mazzei

**Figure 8.**
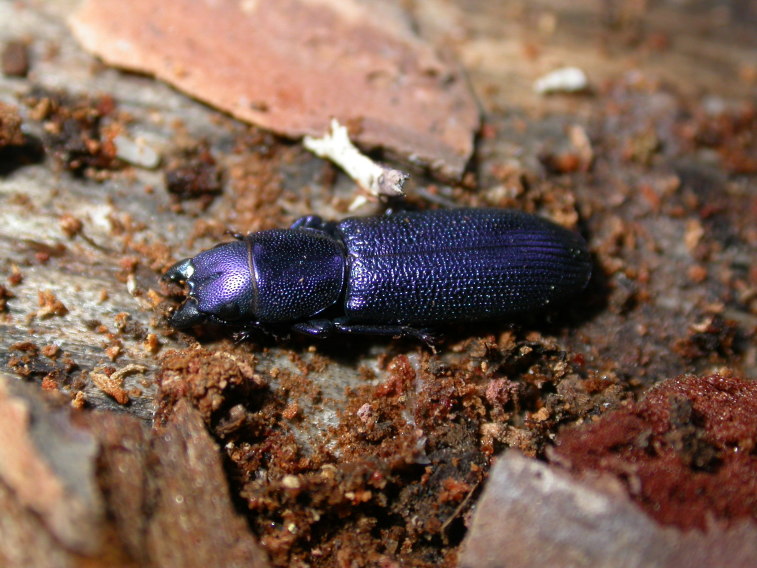
*Temnochila caerulea* (Olivier 1790) – Trogossitidae – photo by Antonio Mazzei

**Figure 9.**
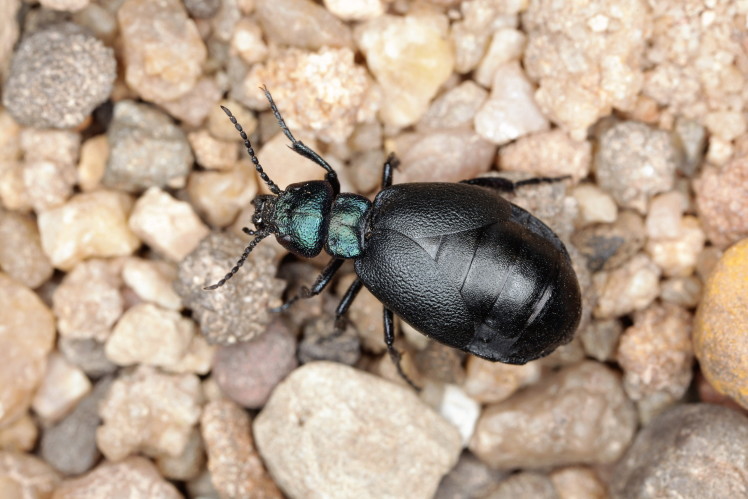
*Meloe decorus* Brandt & Erichson, 1832 – Meloidae – photo by Christoph Benisch – www.kerbtier.de

The Fauna Europaea database contains the scientific names of all living European lands and freshwater animal species, including numerous infra-groups and synonyms. More details about the conceptual background of Fauna Europaea and standards followed are described in the project description papers (Figs 4, 5, 6, 7, 8, 9​).

This data paper covers the Coleoptera content of Fauna Europaea, including 80 Families 12,425 species, 3,663 subspecies and 6,660 (sub)species synonyms. Higher ranks are given below, the species list can be downloaded from the Fauna Europaea portal (see: Data resources).

### Taxa included

**Table:** 

Rank	Scientific Name	Common Name
kingdom	Animalia	
subkingdom	Eumetazoa	
phylum	Arthropoda	
subphylum	Hexapoda	
class	Insecta	
order	Coleoptera	
suborder	Adephaga	
suborder	Archostemata	
suborder	Myxophaga	
suborder	Polyphaga	
infraorder	Bostrichiformia	
infraorder	Cucujiformia	
superfamily	Bostrichoidea	
superfamily	Caraboidea	
superfamily	Chrysomeloidea	
superfamily	Clavicornia	
superfamily	Cleroidea	
superfamily	Cucujoidea	
superfamily	Cupedoidea	
superfamily	Dascilloidea	
superfamily	Dermestoidea	
superfamily	Derodontoidea	
superfamily	Heteromera	
superfamily	Lymexyloidea	
superfamily	Sphaeriusoidea	
superfamily	Tenebrionoidea	
family	Acanthocnemidae	
family	Aderidae	
family	Alexiidae	
family	Anobiidae	
family	Anthicidae	
family	Biphyllidae	
family	Boridae	
family	Bostrichidae	
family	Carabidae	
family	Cerambycidae	
family	Cerylonidae	
family	Chrysomelidae	
family	Ciidae	
family	Clambidae	
family	Cleridae	
family	Coccinellidae	
family	Colydiidae	
family	Corylophidae	
family	Crowsoniellidae	
family	Cryptophagidae	
family	Cucujidae	
family	Cybocephalidae	
family	Dascillidae	
family	Dasytidae	
family	Dermestidae	
family	Derodontidae	
family	Diphyllidae	
family	Dytiscidae	
family	Scirtidae	
family	Endecatomidae	
family	Endomychidae	
family	Erotylidae	
family	Eucinetidae	
family	Gietellidae	
family	Gyrinidae	
family	Haliplidae	
family	Hydroscaphidae	
family	Hygrobiidae	
family	Jacobsoniidae	
family	Kateretidae	
family	Laemophloeidae	
family	Lagriidae	
family	Languriidae	
family	Latridiidae	
family	Lyctidae	
family	Lymexylidae	
family	Melandryidae	
family	Meloidae	
family	Melyridae	
family	Micromalthidae	
family	Monotomidae	
family	Mordellidae	
family	Mycetophagidae	
family	Mycteridae	
family	Nitidulidae	
family	Nosodendridae	
family	Noteridae	
family	Oedemeridae	
family	Passandridae	
family	Phalacridae	
family	Phloeostichidae	
family	Phloiophilidae	
family	Prionoceridae	
family	Prostomidae	
family	Pyrochroidae	
family	Pythidae	
family	Rhipiceridae	
family	Rhipiphoridae	
family	Ripiphoridae	
family	Salpingidae	
family	Scirtidae	
family	Scraptiidae	
family	Serropalpidae	
family	Silvanidae	
family	Sphaeriusidae	
family	Sphaerosomatidae	
family	Sphindidae	
family	Stenotrachelidae	
family	Tenebrionidae	
family	Tetratomidae	
family	Thanerocleridae	
family	Trachypachidae	
family	Trogossitidae	
family	Zopheridae	
subfamily	Agabinae	
subfamily	Agleninae	
subfamily	Agnathinae	
subfamily	Alfieriellinae	
subfamily	Alleculinae	
subfamily	Alticinae	
subfamily	Anamorphinae	
subfamily	Anaspidinae	
subfamily	Anobiinae	
subfamily	Anthicinae	
subfamily	Apotominae	
subfamily	Atomariinae	
subfamily	Bergininae	
subfamily	Bostrichinae	
subfamily	Brachininae	
subfamily	Broscinae	
subfamily	Bruchinae	
subfamily	Calopodinae	
subfamily	Calyptomerinae	
subfamily	Carabinae	
subfamily	Carpophilinae	
subfamily	Cassidinae	
subfamily	Cerambycinae	
subfamily	Ceryloninae	
subfamily	Chaetomalachinae	
subfamily	Chilocorinae	
subfamily	Chlaeniinae	
subfamily	Chrysomelinae	
subfamily	Cicindelinae	
subfamily	Cillaeinae	
subfamily	Clambinae	
subfamily	Clerinae	
subfamily	Coccidulinae	
subfamily	Coelometopinae	
subfamily	Colydiinae	
subfamily	Colymbetinae	
subfamily	Copelatinae	
subfamily	Corticariinae	
subfamily	Corylophinae	
subfamily	Criocerinae	
subfamily	Cryptarchinae	
subfamily	Cryptocephalinae	
subfamily	Cryptophaginae	
subfamily	Cryptophaginae	
subfamily	Cryptophilinae	
subfamily	Cyclosominae	
subfamily	Dacninae	
subfamily	Danaceinae	
subfamily	Dascillinae	
subfamily	Dasytinae	
subfamily	Diaperinae	
subfamily	Dinoderinae	
subfamily	Donaciinae	
subfamily	Dorcatominae	
subfamily	Dryophilinae	
subfamily	Dryptinae	
subfamily	Dytiscinae	
subfamily	Elaphrinae	
subfamily	Encaustinae	
subfamily	Endomychinae	
subfamily	Enopliinae	
subfamily	Epilachninae	
subfamily	Epuraeinae	
subfamily	Ernobiinae	
subfamily	Esarcinae	
subfamily	Eucradinae	
subfamily	Eumolpinae	
subfamily	Eustrophinae	
subfamily	Euxestinae	
subfamily	Galerucinae	
subfamily	Gibbiinae	
subfamily	Gyrininae	
subfamily	Hallomeninae	
subfamily	Harpalinae	
subfamily	Hispinae	
subfamily	Holoparamecinae	
subfamily	Hydroporinae	
subfamily	Hypocoprinae	
subfamily	Korynetinae	
subfamily	Laccophilinae	
subfamily	Lagriinae	
subfamily	Lamiinae	
subfamily	Lamprosomatinae	
subfamily	Latridiinae	
subfamily	Lebiinae	
subfamily	Leiestinae	
subfamily	Lepturinae	
subfamily	Licininae	
subfamily	Lissodeminae	
subfamily	Loricerinae	
subfamily	Lycoperdininae	
subfamily	Lyctinae	
subfamily	Macratriinae	
subfamily	Malachiinae	
subfamily	Melaeninae	
subfamily	Meligethinae	
subfamily	Meloinae	
subfamily	Merophysiinae	
subfamily	Mesocoleopodinae	
subfamily	Murmidiinae	
subfamily	Mycetaeinae	
subfamily	Mycetophaginae	
subfamily	Nacerdinae	
subfamily	Nebriinae	
subfamily	Necydalinae	
subfamily	Nemognathinae	
subfamily	Nitidulinae	
subfamily	Noterinae	
subfamily	Odacanthinae	
subfamily	Oedemerinae	
subfamily	Omophroninae	
subfamily	Oodinae	
subfamily	Orsodacninae	
subfamily	Ortaliinae	
subfamily	Orthoperinae	
subfamily	Palorinae	
subfamily	Panagaeinae	
subfamily	Parandrinae	
subfamily	Patrobinae	
subfamily	Paussinae	
subfamily	Pedilinae	
subfamily	Pelecotominae	
subfamily	Peltinae	
subfamily	Perigoninae	
subfamily	Phalacrinae	
subfamily	Phrenapatinae	
subfamily	Pimeliinae	
subfamily	Platyninae	
subfamily	Pleganophorinae	
subfamily	Polycaoninae	
subfamily	Prioninae	
subfamily	Promecognathinae	
subfamily	Psoinae	
subfamily	Psydrinae	
subfamily	Pterostichinae	
subfamily	Ptilininae	
subfamily	Ptilophorinae	
subfamily	Ptininae	
subfamily	Pyrochroinae	
subfamily	Rhadalinae	
subfamily	Rhysodinae	
subfamily	Ripidiinae	
subfamily	Ripiphorinae	
subfamily	Rypobiinae	
subfamily	Salpinginae	
subfamily	Scaritinae	
subfamily	Scraptiinae	
subfamily	Scymninae	
subfamily	Setariolinae	
subfamily	Siagoninae	
subfamily	Spondylidinae	
subfamily	Steropinae	
subfamily	Sticholotidinae	
subfamily	Synetinae	
subfamily	Tarsosteninae	
subfamily	Telmatophilinae	
subfamily	Tenebrioninae	
subfamily	Tetratominae	
subfamily	Tillinae	
subfamily	Tomoderinae	
subfamily	Toraminae	
subfamily	Trachypachinae	
subfamily	Trechinae	
subfamily	Tritominae	
subfamily	Trogossitinae	
subfamily	Vesperinae	
subfamily	Xenoscelinae	
subfamily	Xyletininae	
subfamily	Zeugophorinae	
subfamily	Zopherinae	
tribe	Abacetini	
tribe	Aciliini	
tribe	Adeliini	
tribe	Adesmiini	
tribe	Adoxini	
tribe	Agabini	
tribe	Akidini	
tribe	Alfieriellini	
tribe	Alphitobiini	
tribe	Amauronioidini	
tribe	Amblicerini	
tribe	Anaspidini	
tribe	Anisodactylini	
tribe	Anthicini	
tribe	Apatini	
tribe	Apenini	
tribe	Apotomini	
tribe	Asclerini	
tribe	Asidini	
tribe	Atomariini	
tribe	Belopini	
tribe	Bembidiini	
tribe	Berginini	
tribe	Bidessini	
tribe	Blaptini	
tribe	Bolitophagini	
tribe	Bostrichini	
tribe	Brachinini	
tribe	Broscini	
tribe	Bruchini	
tribe	Bulaeini	
tribe	Caenoscelini	
tribe	Calleidini	
tribe	Callistini	
tribe	Calopodini	
tribe	Carabini	
tribe	Cassidini	
tribe	Ceratanisini	
tribe	Cerocomini	
tribe	Chilocorini	
tribe	Chlaeniini	
tribe	Cicindelini	
tribe	Clivinini	
tribe	Clytrini	
tribe	Cnemeplatiini	
tribe	Coccidulini	
tribe	Coccinellini	
tribe	Coelometopini	
tribe	Colymbetini	
tribe	Conaliini	
tribe	Copelatini	
tribe	Corsyrini	
tribe	Corylophini	
tribe	Cossyphini	
tribe	Cossyphodini	
tribe	Crypticini	
tribe	Cryptocephalini	
tribe	Cryptophagini	
tribe	Cybistrini	
tribe	Cychrini	
tribe	Cyclosomini	
tribe	Cymbionotini	
tribe	Cymindidini	
tribe	Cynegetini	
tribe	Cynegetini	
tribe	Dalyatini	
tribe	Demetriadini	
tribe	Dendarini	
tribe	Diaperini	
tribe	Dicaelini	
tribe	Ditomini	
tribe	Ditylini	
tribe	Dromiini	
tribe	Dryptini	
tribe	Dyschiriini	
tribe	Dytiscini	
tribe	Elaphrini	
tribe	Elenophorini	
tribe	Endomiini	
tribe	Epicautini	
tribe	Epilachnini	
tribe	Epitragini	
tribe	Eretini	
tribe	Erodiini	
tribe	Esarcini	
tribe	Eumolpini	
tribe	Eurychorini	
tribe	Formicomini	
tribe	Galerucini	
tribe	Gloeosomatini	
tribe	Gyrinini	
tribe	Harpalini	
tribe	Helopini	
tribe	Hydaticini	
tribe	Hydrocanthini	
tribe	Hydroporini	
tribe	Hydrovatini	
tribe	Hygrotini	
tribe	Hyperaspidini	
tribe	Hyphydrini	
tribe	Hypocoprini	
tribe	Hypophloeini	
tribe	Kytorhinini	
tribe	Laccophilini	
tribe	Laccornini	
tribe	Lacnogyini	
tribe	Lagriini	
tribe	Lebiini	
tribe	Leichenini	
tribe	Lestignathini	
tribe	Licinini	
tribe	Lionychini	
tribe	Litoborini	
tribe	Loricerini	
tribe	Luperini	
tribe	Lyctini	
tribe	Lyttini	
tribe	Macrosiagonini	
tribe	Masoreini	
tribe	Megacephalini	
tribe	Melanimini	
tribe	Meloini	
tribe	Methlini	
tribe	Microhorini	
tribe	Microweiseini	
tribe	Microweiseini	
tribe	Mordellini	
tribe	Mordellistenini	
tribe	Morionini	
tribe	Mycetophagini	
tribe	Mylabrini	
tribe	Myrmechixenini	
tribe	Nacerdini	
tribe	Nebriini	
tribe	Nemognathini	
tribe	Nodinini	
tribe	Noterini	
tribe	Notiophilini	
tribe	Notoxini	
tribe	Noviini	
tribe	Odacanthini	
tribe	Oedemerini	
tribe	Omophronini	
tribe	Omphreini	
tribe	Oodini	
tribe	Opatrini	
tribe	Orectochilini	
tribe	Pachybrachini	
tribe	Pachymerini	
tribe	Pachypterini	
tribe	Panagaeini	
tribe	Parmulini	
tribe	Patrobini	
tribe	Paussini	
tribe	Pedinini	
tribe	Pelophilini	
tribe	Pentariini	
tribe	Perigonini	
tribe	Phaleriini	
tribe	Phrenapatini	
tribe	Pimeliini	
tribe	Platynaspidini	
tribe	Platynini	
tribe	Platynotini	
tribe	Platyopini	
tribe	Platyscelini	
tribe	Pogonini	
tribe	Pseudotrechini	
tribe	Psydrini	
tribe	Psylloborini	
tribe	Pterostichini	
tribe	Pycnomerini	
tribe	Rhaebini	
tribe	Rhysodini	
tribe	Ripiphorini	
tribe	Rypobiini	
tribe	Scaphidemini	
tribe	Scaritini	
tribe	Scaurini	
tribe	Scraptiini	
tribe	Scymnini	
tribe	Sepidiini	
tribe	Serangiini	
tribe	Serangiini	
tribe	Sericoderini	
tribe	Sermylini	
tribe	Siagonini	
tribe	Singilini	
tribe	Sinoxylini	
tribe	Somotrichini	
tribe	Sphodrini	
tribe	Stenaliini	
tribe	Stenoderini	
tribe	Stenolophini	
tribe	Stenosini	
tribe	Stenostomatini	
tribe	Stethorini	
tribe	Sticholotidini	
tribe	Stomini	
tribe	Strongyliini	
tribe	Stylosomini	
tribe	Telmatophilini	
tribe	Tenebrionini	
tribe	Tentyriini	
tribe	Teplinini	
tribe	Tetrabrachini	
tribe	Thaneroclerini	
tribe	Trachypachini	
tribe	Trachyscelini	
tribe	Trechini	
tribe	Triboliini	
tribe	Trogoxylini	
tribe	Typhaeini	
tribe	Tytthaspididini	
tribe	Ulomini	
tribe	Xyloperthini	
tribe	Zabrini	
tribe	Zophosini	
tribe	Zuphiini	
subtribe	Acanthoscelidina	
subtribe	Aepina	
subtribe	Amblicerina	
subtribe	Amblystomina	
subtribe	Anillina	
subtribe	Aptinina	
subtribe	Atranopsina	
subtribe	Aulacophorina	
subtribe	Bembidiina	
subtribe	Brachinina	
subtribe	Broscina	
subtribe	Bruchina	
subtribe	Calathina	
subtribe	Calosomatina	
subtribe	Carabina	
subtribe	Caryedonina	
subtribe	Chlaeniina	
subtribe	Cicindelina	
subtribe	Clinidiina	
subtribe	Clivinina	
subtribe	Cymindidina	
subtribe	Diabroticina	
subtribe	Ditomina	
subtribe	Dolichina	
subtribe	Harpalina	
subtribe	Kytorhinina	
subtribe	Lionychina	
subtribe	Luperina	
subtribe	Mastacina	
subtribe	Megacephalina	
subtribe	Molopina	
subtribe	Myadina	
subtribe	Odacanthina	
subtribe	Omoglymmiina	
subtribe	Oodina	
subtribe	Panagaeina	
subtribe	Paussina	
subtribe	Perileptina	
subtribe	Pheropsophina	
subtribe	Poecilina	
subtribe	Pseudomasoreina	
subtribe	Psydrina	
subtribe	Pterostichina	
subtribe	Reicheiina	
subtribe	Rhaebina	
subtribe	Rhysodina	
subtribe	Scaritina	
subtribe	Sphodrina	
subtribe	Synuchina	
subtribe	Tachyina	
subtribe	Trechina	
subtribe	Trechodina	
subtribe	Trichina	
family	Byturidae	

## Temporal coverage

**Living time period:** Currently living.

### Notes

Currently living multicellular, terrestrial and freshwater animals in stable populations, largely excluding (1) rare / irregular immigrants, (2) alien / invasive species, (3) accidental or deliberate releases of exotic (pet)species, (4) domesticated animals, (5) non-native species imported and released for bio-control or (6) non-native species largely confined to hothouses.

## Usage rights

### Use license

Open Data Commons Attribution License

### IP rights notes

Fauna Europaea data are licensed under CC BY SA version 4.0. The property rights of experts over their data is covered under the SMEBD conditions. For more copyrights and citation details see: http://www.faunaeur.org/copyright.php

## Data resources

### Data package title

Fauna Europaea - Coleoptera - 2

### Resource link

http://www.faunaeur.org/Data_papers/FaEu_Coleoptera-2_2.6.2.zip

### Alternative identifiers

http://www.faunaeur.org/experts.php?id=18

### Number of data sets

2

### Data set 1.

#### Data set name

Fauna Europaea - Coleoptera 2 (excl...) version 2.6.2 - species

#### Data format

CSV

#### Number of columns

25

#### Character set

UTF-8

#### Download URL

http://www.faunaeur.org/Data_papers/FaEu_Coleoptera-2_2.6.2.zip

#### Description

**Data set 1.** 

Column label	Column description
datasetName	The name identifying the data set from which the record was derived (http://rs.tdwg.org/dwc/terms/datasetName).
version	Release version of data set.
versionIssued	Issue data of data set version.
rights	Information about rights held in and over the resource (http://purl.org/dc/terms/rights).
rightsHolder	A person or organization owning or managing rights over the resource (http://purl.org/dc/terms/rightsHolder).
accessRights	Information about who can access the resource or an indication of its security status (http://purl.org/dc/terms/accessRights).
taxonID	An identifier for the set of taxon information (http://rs.tdwg.org/dwc/terms/taxonID)
parentNameUsageID	An identifier for the name usage of the direct parent taxon (in a classification) of the most specific element of the scientificName (http://rs.tdwg.org/dwc/terms/parentNameUsageID).
scientificName	The full scientific name, with authorship and date information if known (http://rs.tdwg.org/dwc/terms/scientificName).
acceptedNameUsage	The full name, with authorship and date information if known, of the currently valid (zoological) taxon (http://rs.tdwg.org/dwc/terms/acceptedNameUsage).
originalNameUsage	The original combination (genus and species group names), as firstly established under the rules of the associated nomenclaturalCode (http://rs.tdwg.org/dwc/terms/originalNameUsage).
family	The full scientific name of the family in which the taxon is classified (http://rs.tdwg.org/dwc/terms/family).
familyNameId	An identifier for the family name.
genus	The full scientific name of the genus in which the taxon is classified (http://rs.tdwg.org/dwc/terms/genus).
subgenus	The full scientific name of the subgenus in which the taxon is classified. Values include the genus to avoid homonym confusion (http://rs.tdwg.org/dwc/terms/subgenus).
specificEpithet	The name of the first or species epithet of the scientificName (http://rs.tdwg.org/dwc/terms/specificEpithet).
infraspecificEpithet	The name of the lowest or terminal infraspecific epithet of the scientificName, excluding any rank designation (http://rs.tdwg.org/dwc/terms/infraspecificEpithet).
taxonRank	The taxonomic rank of the most specific name in the scientificName (http://rs.tdwg.org/dwc/terms/infraspecificEpithet).
scientificNameAuthorship	The authorship information for the scientificName formatted according to the conventions of the applicable nomenclaturalCode (http://rs.tdwg.org/dwc/terms/scientificNameAuthorship).
authorName	Author name information
namePublishedInYear	The four-digit year in which the scientificName was published (http://rs.tdwg.org/dwc/terms/namePublishedInYear).
Brackets	Annotation if authorship should be put between parentheses.
nomenclaturalCode	The nomenclatural code under which the scientificName is constructed (http://rs.tdwg.org/dwc/terms/nomenclaturalCode).
taxonomicStatus	The status of the use of the scientificName as a label for a taxon (http://rs.tdwg.org/dwc/terms/taxonomicStatus).
resourceDescription	An account of the resource, including a data-paper DOI (http://purl.org/dc/terms/description)

### Data set 2.

#### Data set name

Fauna Europaea - Coleoptera 2 (excl...) version 2.6.2 - hierarchy

#### Data format

CSV

#### Number of columns

12

#### Character set

UTF-8

#### Download URL

http://www.faunaeur.org/Data_papers/FaEu_Coleoptera-2_2.6.2.zip

#### Description

**Data set 2.** 

Column label	Column description
datasetName	The name identifying the data set from which the record was derived (http://rs.tdwg.org/dwc/terms/datasetName).
version	Release version of data set.
versionIssued	Issue data of data set version.
rights	Information about rights held in and over the resource (http://purl.org/dc/terms/rights).
rightsHolder	A person or organization owning or managing rights over the resource (http://purl.org/dc/terms/rightsHolder).
accessRights	Information about who can access the resource or an indication of its security status (http://purl.org/dc/terms/accessRights).
taxonName	The full scientific name of the higher-level taxon
scientificNameAuthorship	The authorship information for the scientificName formatted according to the conventions of the applicable nomenclaturalCode (http://rs.tdwg.org/dwc/terms/scientificNameAuthorship).
taxonRank	The taxonomic rank of the most specific name in the scientificName (http://rs.tdwg.org/dwc/terms/infraspecificEpithet).
taxonID	An identifier for the set of taxon information (http://rs.tdwg.org/dwc/terms/taxonID)
parentNameUsageID	An identifier for the name usage of the direct parent taxon (in a classification) of the most specific element of the scientificName (http://rs.tdwg.org/dwc/terms/parentNameUsageID).
resourceDescription	An account of the resource, including a data-paper DOI (http://purl.org/dc/terms/description)

## Supplementary Material

Supplementary material 1 FaEu Coleoptera 2 stats Data type: png
Brief description: This is a high-resolution version of Figure 3.
File: oo_32851.png Yde de Jong & Paolo Audisio

## Figures and Tables

**Table 1.** Responsible specialists per family in Coleoptera

FAMILY	NUMBER OF SPECIES IN FAEU (in case of estimated gaps: potential numbers in brackets)	SPECIALIST(S)
Acanthocnemidae	1	Gianfranco Liberti
Aderidae	27	Gianluca Nardi
Alexiidae	32 (≈ 40)	Wioletta K. Tomaszewska
Anobiidae	419 (≈ 430)	Petr Zahradnik
Anthicidae	314	Gianluca Nardi
Biphyllidae	5	Josef Jelínek (resigned)
Boridae	1	Xavier Vazquez-Albalate
Bostrichidae	42 (≈ 45)	Gianluca Nardi
Bothrideridae	106 (≈120)	Adam Slipinski
Byturidae	3	Josef Jelínek (resigned)
Carabidae	3738 (≈ 3900)	Augusto Vigna Taglianti
Cerambycidae	677 (≈ 680)	Gianfranco Sama
Cerylonidae	14	Adam Slipinski
Chrysomelidae	1758 (≈ 1800)	Maurizio Biondi, Ron Beenen, Michael Schmitt, Renato Regalin, David Sassi, Stefano Zoia, Horst Kippenberg & Marcello Franco Zampetti
Ciidae	76 (≈ 80)	Josef Jelínek & Paolo Audisio
Clambidae	22	Ivan Löbl
Cleridae	68 (≈ 70)	Roland Gerstmeier
Coccinellidae	215 (≈ 220)	Claudio Canepari
Corylophidae	37 (≈ 40)	Paolo Audisio
Crowsoniellidae	1	Paolo Audisio
Cryptophagidae	257 (≈ 260)	Carlos Otero
Cucujidae	6 (≈ 8)	Adam Slipinski
Cybocephalidae	26 (≈ 30)	Josef Jelínek & Paolo Audisio
Dascillidae	381 (390)	Manfred Jäch
Dermestidae	197 (≈ 200)	Roustem D. Zhantiev
Derodontidae	5	Jirí Háva
Dytiscidae	375 (≈ 400)	Anders Nilsson (first release), Saverio Rocchi & Fabio Cianferoni (future updating)
Endecatomidae	1	Gianluca Nardi
Endomychidae	79 (≈ 80)	Wioletta K. Tomaszewska
Erotylidae	29	Piotr Wegrzynowicz
Eucinetidae	8	Paolo Audisio
Gietellidae	2	Gianfranco Liberti
Gyrinidae	17	Paolo Mazzoldi
Haliplidae	34	Saverio Rocchi & Fabio Cianferoni
Hydroscaphidae	2	Ivan Löbl
Hygrobiidae	1	Anders Nilsson (first release), Saverio Rocchi & Fabio Cianferoni (future updating)
Jacobsoniidae	2	Ivan Löbl
Kateretidae	29 (30)	Paolo Audisio & Josef Jelínek
Laemophloeidae	29	Adam Slipinski
Languriidae	14	Piotr Wegrzynowicz
Latridiidae	192 (≈ 200)	Wolfgang H. Rucker
Lyctidae	13	Gianluca Nardi
Lymexylidae	3	Paolo Audisio
Malachiidae	327 (≈ 330)	Robert Constantin
Melandryidae	53	Nikolai Nikitsky
Meloidae	181 (≈ 185)	Marco Alberto Bologna
Melyridae	18	Gianfranco Liberti
Micromalthidae	1	Paolo Audisio
Monotomidae	34	Josef Jelínek & Paolo Audisio
Mordellidae	256 (≈ 270)	Jan Horak
Mycetophagidae	31	Nikolai Nikitsky
Mycteridae	3	Paolo Audisio
Nitidulidae	248 (≈ 250)	Paolo Audisio & Josef Jelínek
Nosodendridae	1	Jiri Hava
Noteridae	4	Anders Nilsson (first release), Saverio Rocchi & Fabio Cianferoni (future updating)
Oedemeridae	93 (≈ 95)	Xavier Vazquez-Albalate
Passandridae	1	Adam Slipinski
Phalacridae	56	Zdenek Svec
Phloeostichidae	1	Adam Slipinski
Phloiophilidae	1	Gianfranco Liberti
Prionoceridae	1	Gianfranco Liberti
Prostomidae	1	Paolo Audisio
Pyrochroidae	9	Gianluca Nardi
Pythidae	5	Xavier Vazquez-Albalate
Rhipiceridae	2	David Kral
Ripiphoridae	17	Federica Turco & Marco Alberto Bologna
Salpingidae	19	Xavier Vazquez-Albalate
Scirtidae	94	Maciej Sapiejewski (deceased), proposed follow-up Rafal Rita
Scraptiidae	102 (≈ 110)	Jan Horak
Silvanidae	40	Adam Slipinski
Sphaeriusidae	3	Ivan Lobl
Sphindidae	4	Josef Jelínek (resigned)
Stenotrachelidae	2	Paolo Audisio
Tenebrionidae	1392 (≈1400)	Simone Fattorini
Tetratomidae	10	Nikolai Nikitsky
Thanerocleridae	1	Roland Gerstmeier
Trachypachidae	1	Saverio Rocchi & Fabio Cianferoni
Trogossitidae	25	Jan Kolibac
Zopheridae	128 (≈ 130)	Adam Slipinski
