# Supplementary figures and images for: Fauna Europaea: Coleoptera 2 (excl. series Elateriformia, Scarabaeiformia, Staphyliniformia and superfamily Curculionoidea)
Source: Biodivers Data J. 2015 Apr 9;(3):e4750. doi: 10.3897/BDJ.3.e4750 (PMC4399155; doi:10.3897/BDJ.3.e4750)

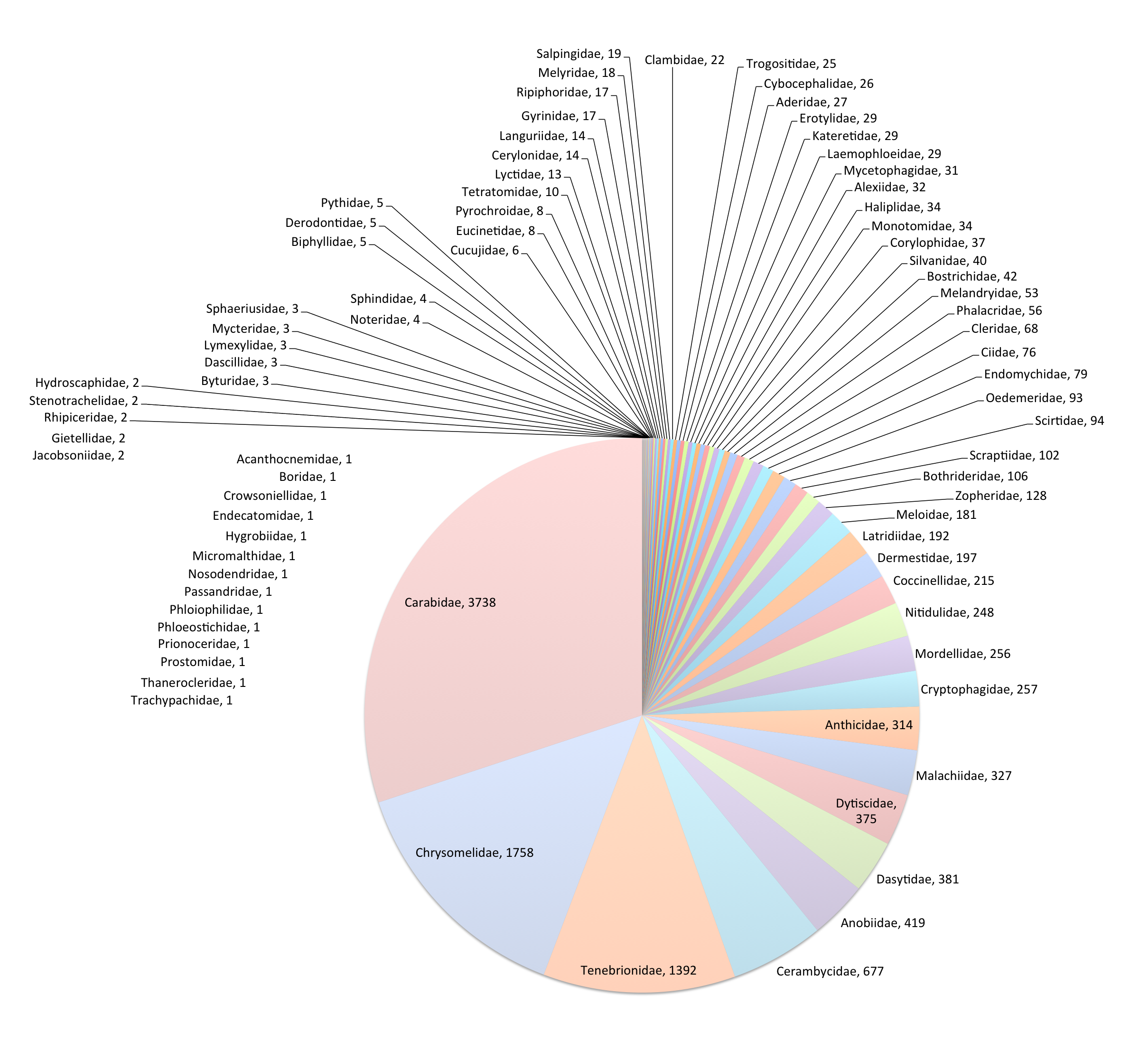

Supplement: Supplementary material 1 — FaEu Coleoptera 2 stats [file biodiversity_data_journal-3-e4750-s001.png]
